# The antioxidant, antidiabetic, and antihyperlipidemic effects of the polyphenolic extract from *Salvia blancoana* subsp. *mesatlantica* on induced diabetes in rats

**DOI:** 10.1186/s40643-024-00769-1

**Published:** 2024-06-26

**Authors:** Souad Maache, Hassan Laaroussi, Najoua Soulo, Ghizlane Nouioura, Nabil Boucetta, Mohammed Bouslamti, Hamza Saghrouchni, Yousef A. Bin Jardan, Samir Ibenmoussa, Mohammed Bourhia, Badiaa Lyoussi, Ilham Elarabi

**Affiliations:** 1 Laboratory of Natural Substances, Pharmacology, Environment, Modeling, Health, and Quality of Life (SNAMOPEQ), Faculty of Sciences Dhar El Mahraz, Sidi Mohamed Ben Abdellah University, Fez, Morocco; 2 Medical Analysis Laboratory Sais, Fez, Morocco; 3 Department of Biotechnology, Institute of Natural and Applied Sciences, Çukurova University, 01250 Balcalı Adana, Türkiye; 4 Department of Pharmaceutics, College of Pharmacy, King Saud University, P.O. Box 11451, Riyadh, Saudi Arabia; 5 Laboratory of Therapeutic and Organic Chemistry, Faculty of Pharmacy, University of Montpellier, 34000 Montpellier, France; 6 Laboratory of Biotechnology and Natural Resources Valorization , Faculty of Sciences, Ibn Zohr University, 80060 Agadir, Morocco; 7 Laboratory of Chemistry-Biochemistry, Environment, Nutrition, and Health, Faculty of Medicine and Pharmacy, University Hassan II, B. P. 5696, Casablanca, Morocco

**Keywords:** Anti-diabetic, Antioxidant, Extract, Chemical compounds, *Salvia blancoana *subsp*. mesatlantica*

## Abstract

Currently, several studies have demonstrated the benefits of medicinal plants in managing type 2 diabetes. In this work, we evaluated the beneficial effects of the polyphenolic extract (PESB) from *Salvia blancoana* subsp*. mesatlantica* in the management of hypercaloric-feeding and small-dose alloxan-brought type 2 diabetes in rats. We analyzed the chemical constituents of the extract, including flavones and flavonols content, to understand its biological action. The antioxidant activities were evaluated by total antioxidant action, scavenging effect of the free radical DPPH, and reducing power. The obtained results showed that the value of TFC was estimated at 31.90 ± 0.34 mgEQ/g in the PESB extract. The total antioxidant capacity was estimated at 593.51 ± 4.09 mg (EAA)/g, the value of DPPH IC_50_ was 7.3 ± 0.00 μg/mL, and the value of EC_50_ of reducing power was estimated at 6.43 ± 0.01 μg/mL. In total, 14 phenolic compounds were identified and the naringin was the most dominant (63.19%) while the vanillin was the less recorded (0.10%). Serum glucose decreased significantly (p < 0.05) in rats given PESB (100 mg/kg) after four weeks. Glibenclamide (GLB) and PESB reduced HbA1c and increased plasma insulin in diabetic rats, restoring HOMA-β and HOMA-IR levels to near-normal. Additionally, diabetic rats treated with GLB or PESB showed statistically equivalent results to those of non-diabetic rats regarding hepatic enzymes, renal and lipid markers, as well as cardiovascular indices. The weight loss was significantly lower in diabetic rats receiving a dose of PESB (100 mg/kg), and GLB compared to corresponding untreated diabetic rats (p < 0.01). PESB and GLB showed a prominent protective function in the pancreas, liver, and kidney tissues. This investigation demonstrates the capacity of extracts from leaves of *S. blancoana* subsp. *mesatlantica* to manage diabetes mellitus due to their richness in a wide range of bioactive compounds. Therefore, more investigations are required to estimate the safety of the plant use.

**Graphical Abstract:**

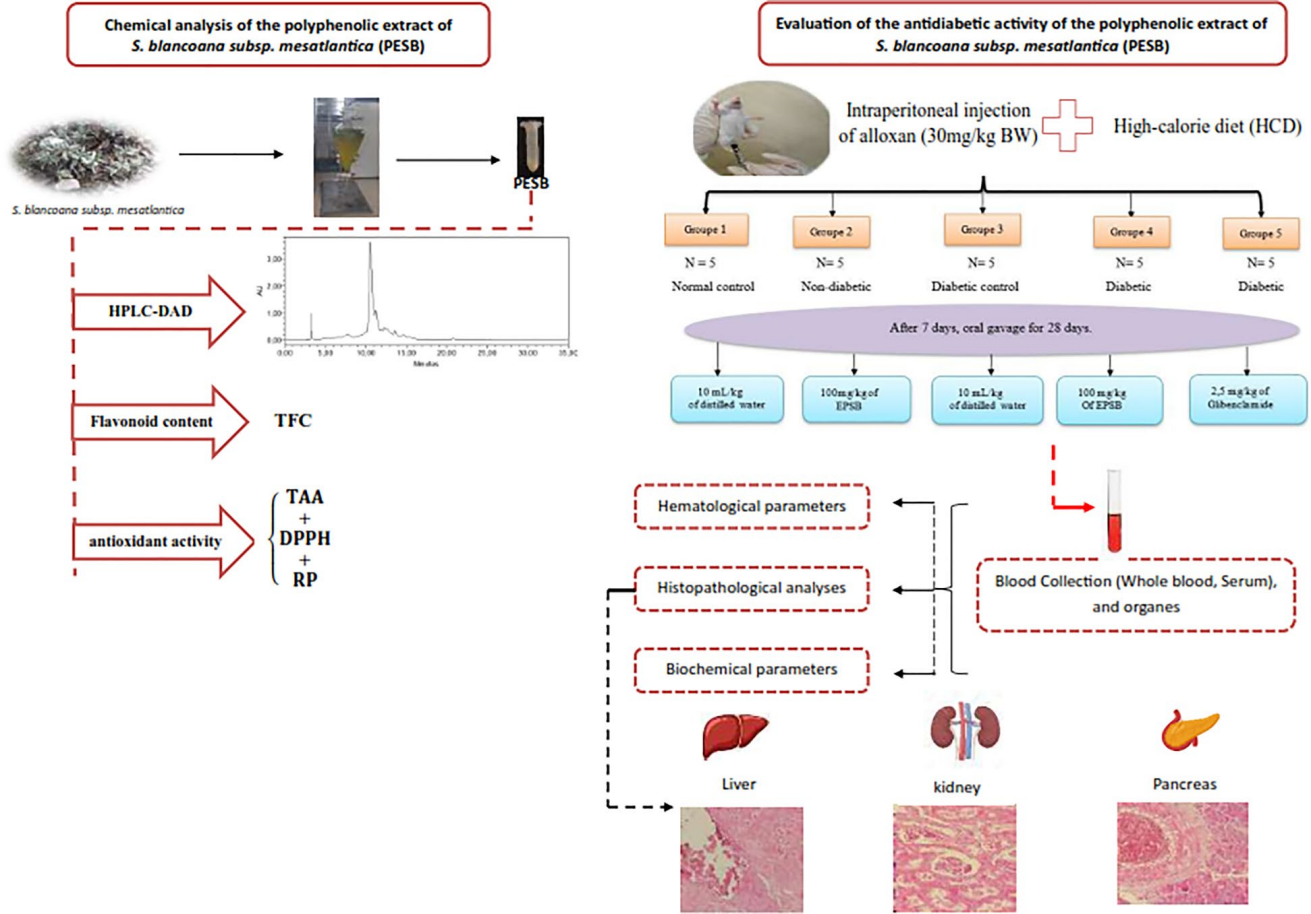

## Introduction

The term “diabetes mellitus” labels a broad range of metabolic diseases marked by a malfunction in the management of glucose, which raises blood glucose levels (Raoufi et al. 2015; Poznyak et al. 2020). The worldwide classification of diseases and several nations use the Latin word diabetes mellitus (Liu et al. 2020). When insulin fails, the fast breakdown of fats that results in severe hyperglycemia can cause acetone toxicity that damages the kidneys, heart, or retina (Sabari et al. 2023). This illness also manifests as polyuropolydipsic syndrome, a set of symptoms unique to diabetes (Chang et al. 2018; Rachdaoui 2020; Drissa 2021). The progressive condition recognized as type 2 diabetes (T2D) is marked by a steady and gradual decline in the role of pancreatic β cells (Wright et al. 1998; Dludla et al. 2023). Long-term issues include persistent injuries, organ failure, eyesight loss, kidney illnesses, and peripheral neuropathy with a risk of foot ulcers, or even amputation are linked to chronic consequences of diabetes (Care 2020; The Expert Committee on the Diagnosis and Classification of Diabetes Mellitus 2003).

As one of the chief roots of death globally, diabetes is currently one of the most pervasive and dangerous health issues (Afrisham et al. 2015). Numerous research has reported that oxidative stress and inflammation are two disorders that are linked to diabetes (Román-Pintos et al. 2016; Asmat et al. 2016). Equally, current investigations have evaluated the intensity of oxidative anxiety in participants impacted by diabetes mellitus, while other investigations have addressed the mechanisms (molecular and pathway) relating diabetes mellitus and oxidative stress (Ighodaro 2018; Yaribeygi et al. 2020). Enhanced oxidative stress in participants with diabetes mellitus is caused by an inequity between the body's biological antioxidant system and the generation of free oxygen radicals, which accelerates the development and worsening of diabetic problems (Assadi et al. 2021). According to a 2017 study by the International Federation of Diabetes (IDF), the total of individuals impacted by diabetes is rising exponentially, with over 425 million estimated to be affected by the disease by 2045. A sedentary lifestyle, eating foods high in energy, obesity, and longer life expectancies are some of the causes of this rise (Yajnik 2001). The primary cause of metabolic risk factors linked to cardiovascular disease, hypercholesterolemia, hypertension, hyperglycemia, type 2 diabetes, and several cancers is a hypercaloric diet (HCD) (Singla et al. 2010; Ousaaid et al. 2020).

The usage of insulin, medication, and particular diets are among the therapeutic alternatives that have been accessible for the control of diabetes for the past few decades (Tahrani et al. 2016; Thrasher 2017). Even if there are multiple medications that have antidiabetic effects in diverse ways, getting the best possible treatment response is still difficult (Abd Rashed and Rathi 2021). Additionally, research on ancient herbal medicines has been restored due to the increased interest in alternative medication (Amaeze et al. 2018; Najmi et al. 2022). Many florae extracts are beneficial in controlling glucose metabolism in the latest clinical reports (Singab et al. 2014; Anderson et al. 2016; Lee et al. 2021). Plant items with high concentrations of flavonoids, glycosides, phenolic composites, terpenoids, alkaloids, and coumarins are effective in controlling the metabolism of carbohydrates (Assadi et al. 2021).

Globally, there exists a vast array of therapeutic plants, especially Salvia species gaining widespread recognition for their remarkable health advantages (Porres-Martínez et al. 2017; Abd Rashed and Rathi 2021; Uysal et al. 2023). About 900 species of the Lamiaceae family’s genus Salvia can be found throughout the world, primarily in the Mediterranean mountains, South-East Africa, Caribbean (Central America), and South zones of America (Longaray Delamare et al. 2007). Several phenolic metabolites, produced by Salvia species, have enticed a lot of consideration because of their potential benefits as antioxidants, antimicrobials, antidiabetics, neuroprotectors, anti-inflammatory agents, and even cytotoxic agents (Lu and Yeap Foo 2001; Zengin et al. 2018). Past findings have demonstrated the critical function that flavonoids and phenolic compounds play in inhibiting both α-amylase and α-glucosidase (Moradi-Afrapoli et al. 2012; Asghari et al. 2015). In vivo confirmation of the hypoglycemic effects of some Salvia species has also been obtained (Abd Rashed and Rathi 2021).

*S. blancoana* subsp*. mesatlantica*, also known as *S. lavandulifolia subsp. mesatlantica*, is one of the most widely utilized sage species in regions located in the Atlas (middle zones). The hypoglycemic role of this vegetable has long been recognized, particularly in the treatment of diabetes. With lovely malva blue blooms and a preference for the sand-calcareous soils of mountainous regions situated at elevations ranging from 300 to more than 1000 m above sea level, this woody shrub has a long lifespan and typically reaches a height of 17–100 cm (Cutillas et al. 2017). Research has indicated that the aqueous extract derived from the decoction of *S. lavandulifolia* Vahl leaves exhibits both an antihyperglycemic and an inhibitory impact on the α-amylase enzyme in normal rats that have been overfed starch or D-glucose (Remok et al. 2023). These results underline the significance of more research on this exciting subject and show the therapeutic potential of the Salvia species, notably *S. lavandulifolia* Vahl, in the regulation of diabetes.

To explore the probable course of action and assess the anti-diabetic benefits of the polyphenolic PESB extract from *S. blancoana subsp. mesatlantica*, a thorough analysis was necessary. This work used a type 2 diabetic rat group created by a low dosage of alloxan and fed a hypercaloric diet to examine the impacts of PESB on blood sugar regulation, resistance to insulin, metabolism of lipids, and liver biomarkers (Raoufi et al. 2015; Vellai et al. 2018). Additionally, the investigation contrasted the outcomes with those attained with Glibenclamide, a reference medication known for its effectiveness in reducing blood glucose amounts by inducing the release of insulin synthesized in beta cells.

As far as we are aware, this study is the primary to assess this product's anti-diabetic qualities from *S. blancoana* subsp. mesatlantica, opening up new avenues for diabetes.

## Materials and methods

### Plant materials

In June 2022, the aerial sections of *Salvia blancoana* subsp. *mesatlantica* (SB) were collected in Immouzer Kandar, situated in the heart of the Middle Atlas in Morocco. The precise taxonomic naming of this herb was conducted by the Department of Plant Ecology and Botany at the Mohammed V University Scientific Institute in Rabat. A reference specimen was documented in the institute's herbarium, identified as RAB 112040. The leaves were then dried in a shaded area at room temperature and processed into a powder using an electric grinding tool.

### Preparation of polyphenolic extract

There were 100 g of SB powder utilized. To do the extraction, 300 mL of methanol were heated to 50 °C for 3 h. Via a rotational evaporator, the preparation was extracted from the targeted extract after the maceration process. Subsequently, the obtained extract was suspended in water (500 mL) and underwent three extraction stages, each of which was repeated three times: first with 200 mL of hexane, followed by 200 mL of chloroform to remove chlorophyll and caffeine residues, and finally with 200 mL of ethyl acetate. Followed by evaporation of the reduced pressure ethyl acetate (Ho et al. 1992; Slighoua et al. 2021). The yield of this polyphenolic extract is 1.86% ± 0.2.

This residue has been carefully preserved in the dark and kept in the freezer for later use.

### Chemical analysis of the extract

#### Flavones and flavonols content (TFF)

The quantification of flavones and flavonols was conducted as follows: 500 μl of each prepared sample was mixed with a solution containing AlCl3 (2% at 500 μl) (Tepal 2016). After 1 h of incubation, the absorption was realized at 420 nm. The experiments were performed for triplet assays and the scores were stated as the average ± SD. Further, values of contained flavones and flavonols were stated in milligrams unit of quercetin corresponding to gram of the herb trial (mg of QE/g) (Bakour et al. 2018; Laaroussi et al. 2020).

#### Identification of polyphenolics by HPLC–DAD

The analysis of polyphenols was conducted via high-performance liquid chromatography (HPLC) equipped by UV detector (range 210 to 400 nm) (Anticona et al. 2022). The separation of polyphenols by HPLC was performed on a reverse-phase (C18) column with features of 4 mm × 25 cm, a particle dimension estimated at 5 μm, and 1 mL/min flow rate. Further, a trinity-mobile form consisting of methanol, acetonitrile, and water was used. The row temperature was maintained at 30 °C, while the injection volume was 40 μL. After the extract was prepared, it was filtered via microfilters (0.45 μm) at a concentration of 50 mg/mL. Detection was performed using spectrophotometer at 280 nm. To identify the compounds, we compared their retention times and UV spectra to established standards (Hbika et al. 2022; Loukili et al. 2022).

### Evaluation of antioxidant activity in vitro

#### Total antioxidant activity (TAA)

The TAA of the PESB sample was assessed using the phosphomolybdate procedure depicted by Prieto et al. (1999). One milliliter of the reactive solution (sodium phosphate (28 mM), sulfuric acid (6 M), and ammonium molybdate (4 mM)) was mixed with PESB (50 μL), and the obtained combination was raised for 90 min at 95 °C in a double boiler. Subsequently, the absorbance (optical density) was assessed at a wave with 695 nm, while the ascorbic acid was considered as the standard. At the end, the recorded scores were stated as milligrams (mg) of ascorbic acid (AA) equivalent (EAA) per g of the used sample (mg EAA/g).

#### Scavenging of the Free Radical DPPH

The procedure designated by Miguel et al. was employed to determine the scavenging action of DPPH radicals (Miguel et al. 2014). In brief, 50 μl of each used sample was interspersed with 875 μl mixture of DPPH (2, 4%), and the absorbance is read at 517 nm. The linear regression equation was employed to graphically calculate the IC_50_ inhibitory concentration, corresponding to the extract concentration that inhibits 50% of the initial substrate concentration. The experiments were conducted in three assays, and the recorded scores were communicated as an average of ± SD (unit mg/mL).

#### Reducing power (RP)

The study involved assessing the reducing power (RP) of the PESB utilizing the iron reduction manner outlined by Oyaizu (1986). Further, 50 μl of the previously diluted samples were interspersed with phosphate buffer (250 μl) and 1% of potassium ferricyanide at a concentration of 250 μl. The resulting combination was then blocked and raised in darkness for 20 min at 50 °C in a double boiler. Additionally, 10% trichloroacetic acid (250 μl) was included along to distilled water (250 μl) and 0.1% ferric chloride (60 μl). Finally, the absorbance (optical density) was assessed at 700 nm.

### Induction of diabetes mellitus and experimental design

#### Experimental animals

We obtained a set of adult male Wistar strain rats, pondering between 190 and 210 g, in collaboration with the animal husbandry center of the Faculty of Science at Sidi Mohamed Ben Abdallah University in Fez. These groups were individually housed in metabolic cages with standardized parameters including temperature, humidity, and 12 h (1/2 day) light/12 h (1/2 day) darkness rotation we reined. Before the start of the study, a 7-day acclimation period was observed to let the rats adjust to their new circumstances.

The welfare and operation of tested animals have been subjected to internationally recognized ethical standards for the exploit of laboratory faunae. Our study procedures have been consented to the official animal protection committee, and we have strictly adhered to the ethical guidelines in place. This compliance is attested by the registration number of our ethical approval: L.20.USMBA-SNAMOPEQ 2020-03.

#### Experimental protocol

In this experimental study on diabetes, the selected rats were divided into five clusters. Then, each prepared group contained five animals (n = 5). The first two groups comprised non-diabetic rats: The first group referred to as NC, contains normal rats (control) fed with a basic diet (this group received only water (distilled) (10 mL per kg of body weight) via gavage). The second group (2) (PESB) contained normal rats and was fed with a basic regime, but they were given 100 mg per kg (of body weight) of polyphenolic extract of SB via gavage.

Groups 3 through 5 were given a little dosage of alloxan to induce type 2 diabetes, and for three months they were given a high-calorie diet (HCD) heavy in fats and carbohydrates, such as peanuts, baked goods, and a beverage with a 10% d-glucose solution. Subsequently, they were inserted with one prescription of a newly fixed solution of alloxan intraperitoneally (30 mg of the product to each kg of body weight of the studied rat in a cold citrate–phosphate (C = 0.1 mM) buffer and pH fixed at 4.5) (Huang et al. 2015). An equivalent volume of citrate buffer was intraperitoneally injected into the control rats. As a precaution, the experimental rats were given a solution of glucose at 10% after a period of 6 h to prevent hypoglycemia (Petchi et al. 2013). Hyperglycemia was measured using a glucometer with reactive strips, and group of rats with abstinence glucose levels exceeding 200 mg/dl were classified as diabetic (Liu et al. 2008).

The details of the groups are as follows:

Group 3 (DC): diabetic control without any treatment.

Group 4 (D + PESB): treatment with 100 mg of polyphenolic extract per each kg of body weight during a period of 4 weeks, starting 7 days after Alloxan injection.

Group 5 (D + Gliben): treatment with reference hypoglycemic drug, of 2.5 mg of Glibenclamide per each kg of body weight of rat for 4 weeks, starting 7 days after Alloxan injection.

#### Biochemical evaluation

Serum trials from tested animals were tested for fasting blood sugar, glycated hemoglobin (HbA1c), enzymes of the liver (aspartate aminotransferase (AST), alkaline phosphatase (ALP), alanine aminotransferase (ALT), and lactate dehydrogenase (LDH)), lipid profile (triglycerides (TG), total cholesterol (TC), high-density lipoproteins (HDL-C), and lipoprotein with low-density (LDL-C)), and parameters of the kidney (urea, total proteins, creatinine, sodium (Na^+^), potassium (K^+^), uric acid, and chloride (Cl^−^)). For samples of urine, tests were performed to measure creatinine, urea, sodium (Na^+^), uric acid, potassium (K^+^), and chloride (Cl^−^).

Plasma insulin levels were determined using the insulin radioimmunoanalysis (RIA) method in rats.

The evaluation of homeostasis parameters, such as the HOMA-IR index (Eq. (1)) and the HOMA-β index (Eq. (2)), was conducted based on the formulas explained by Matthews et al. (1985).

The equations used to calculate these indices are as follows:

1 $${\text{HOMA}} - {\text{IR}}\, = \,{\text{Glucose }}\left( {{\text{mmol L}}^{{ - {1}}} } \right)\, \times \,{\text{Insulin }}(\mu {\text{L}}) \, /{ 22}.{5}$$

2 $${\text{HOMA}} - \beta \, = \,{\text{Insulin }}(\mu {\text{L}})\, \times \,{2}0/ \, \left( {{\text{Glucose }}\left( {{\text{mmol L}}^{{ - {1}}} } \right){-\!\!-}{3}.{5}} \right)$$

The cardiovascular risk index (abbreviation CVRI), atherogenic index (referred to as AI), and coronary artery risk index (referred to as CRI) were estimated utilizing the ensuing formulas (Erejuwa et al. 2016):

AI = LDL/HDL of cholesterol; CRI = total cholesterol/ HDL; CVRI = triglycerides/HDL of cholesterol.

#### Body weight

We conducted weekly weighing of the rats in each group throughout the duration of the experiment using an electronic scale.

### Histological examination of pancreatic, hepatic, and renal tissues

Sections of each pancreatic tissue, liver, and kidney taken from test and trial rats were settled in 10% buffered formol and included in paraffin. The segments were then colored with hematoxylin–eosin (HE) and examined under an optical microscope (Bakour et al. 2018).

### Statistical analysis

We used a one-way test of variance (ANOVA), then followed by the Tukey statistical test to compare groups. The obtained results were displayed as the mean ± SD. The significant results were considered at 0.05.

## Results

### Antioxidant content and antioxidant activities

The antioxidant effects and content in vitro assays of *S. blancoana* subsp. *mesatlantica* polyphenolic extract (PESB) were described in Table 1. The obtained findings revealed that the polyphenolic extract contains 6.10 ± 0.04 mg of quercetin equivalent (QE)/g. Furthermore, PESB extract was found to have a total antioxidant capacity of 593.51 ± 4.09 mg of ascorbic acid equivalent (EAA)/g. In terms of free radical scavenging effect (DPPH), the value of IC_50_ was estimated at 7.3 ± 0.00 μg/mL. Moreover, the value of the EC_50_ of reducing power (RP) was estimated at 6.43 ± 0.01 μg/mL.

**Table 1:** Antioxidant content and antioxidant activities in vitro of *Salvia blancoana* subsp. *mesatlantica* polyphenolic extract (PESB)

	TFF (mgEQ/g)	TAC (mgEAA/g)	DPPH(IC_50_) µg/mL	RP(EC_50_) µg/mL
PESB	31.90 ± 0.34	593.51 ± 4,09	7.3 ± 0.00	6.43 ± 0.01

The data is expressed as an average ± SD. *TFF* flavones and flavonols, *TAC* total antioxidant capacity, *DPPH* 2.2-diphenyl-1-picrylhydrazyle, *RP* reducing power, *mgEQ/g* mg quercetin equivalent/g, *mgEAA/g* mg ascorbic acid equivalent /g

### Identification of polyphenolic compounds

The chemical compounds of the polyphenolic extract from the leaves of *S. blancoana* subsp. *mesatlantica* were identified via high-performance liquid chromatography coupled with diode detection (HPLC–DAD). The identified compounds from the symmetrical peaks are described in Table 2. The recorded results displayed that the area (%) of polyphenols are variable in the polyphenolic extract. In total, 14 phenolic compounds were identified. Naringin was the most dominant followed by cinnamic acid, rutin hydrate, and sinapic acid. The less recorded compounds were kaempferol, rutin, salicylic acid, and vanillin.

**Table 2:** Identified phenolic compounds with HPLC–DAD from polyphenolic extract of *Salvia blancoana* subsp.* mesatlantica*

Phenolic compounds	Formula	Area (%)
Salicylic acid	C_7_H_6_O	0.18
Vanillic acid	C_8_H_8_O_4_	1.25
Vanillin	C_8_H_8_O_3_	0.1
3hydroxy benzoïc acid	C_3_H_6_O_3_	3.89
Naringin	C_27_H_32_O_14_	63.19
Cinnamic acid	C_9_H_8_O_2_	15.39
p-coumaric acid	C_9_H_8_O_3_	3.14
Sinapic acid	C_11_H_12_O_5_	4.43
Rutin hydrate	C_27_H_32_O_17_	5.12
Succinic acid	C_4_H_6_O_4_	2.05
Rutin	C_27_H_30_O_16_	0.26
Quercetin	C_15_H_10_O_7_	0.67
Kaempferol	C_15_H_10_O_6_	0.32
Apigenin	C_15_H_10_O_5_	1.25

### Effects of SB polyphenolic extract in vivo

#### Hypoglycemic properties of SB polyphenolic extract (PESB)

The impact of each day's oral remedy by PESB of *S. blancoana* subsp*. mesatlantica* on the level of glucose in the blood of diabetic and nondiabetic rats is exhibited in Table 3. In the ongoing study, after four weeks of treatment, a very significant and progressive decrease (with *p* < 0.05) in glucose from serum of blood was observed in the subjects receiving PESB (100 mg/kg) and 2.5 mg/kg of body weight of Glibenclamide.

**Table 3:** Influence of PESB from *Salvia blancoana* subsp. *mesatlantica* (oral and daily treatment) on the level of glucose in the blood of both diabetic and control rats

Period	NC	PESB	DC	D + PESB	D + Gliben
0-Day	84.16 ± 17.75^*^d	105.63 ± 4.42^***^c	403.7365.23^*^a	401.13 ± 11.28^***^a	365.00 ± 10.14^***^b
10-Days	84.16 ± 10.05^*^d	105.63 ± 3.77^***^c	403.73 ± 92.50^*^a	401.13 ± 11.69^***^a	365 ± 15.06^***^b
20-Days	88.60 ± 7.52^*^d	96.66 ± 4.79^**^d	431.60 ± 93.48^**^a	293.80 ± 13.30^**^b	251.23 ± 17.08^**^c
30-Days	90.33 ± 11.10^*^c	88.166 ± 3.35^*^c	493.76 ± 68.15^***^a	176.33 ± 11.80^*^b	99.97 ± 2.62^*^c

a > b > c > d (significant difference): comparison between treatments (groups of rats); *** > ** > *(significant difference): comparison between experimental periods for the same group. *NC*: normal rats (control), *PESB*: normal + PESB (100 mg/kg), *DC* control rats with Diabetes, *D + PESB* diabetic + 100 mg/kg PESB, *D + GLB* diabetic + Glibenclamide

The plasma insulin and glycated hemoglobin (HbA1c) values in the test and test groups of rats are displayed in Table 4. Further, there was a discernible ascent in HbA1c in the group of rats with diabetes as compared to the rat group's control. Moreover, compared to rats in control groups, diabetic rats showed signs of insulin insufficiency. However, after the diabetic rats received GLB and PESB orally, there was a notable decline in HbA1c levels and an upsurge in levels of insulin in plasma. Furthermore, the function of beta cells (pancreatic) and resistance to insulin markers, HOMA-β and HOMA-IR, recovered to nearly normal levels in diabetic rats given GLB and PESB treatment, as shown in Table 4. This improvement shows how the PESB extract helps diabetic rats' glycemic and hormonal markers return to normal.

**Table 4:** Effect of PESB and GLB on insulin in serum, HOMA-β, HOMA-IR, and HbA1c in both diabetic and control rats

Experimental groups	Insulin (serum) (mIU/L)	HOMA-IR	HOMA-β	HbA1c (%)
NC	14.67 ± 0.65	1.51 ± 0.07	123.85 ± 9, 77	4.48 ± 0.05
PESB	15.03 ± 0.18	1.53 ± 0.04	127.54 ± 1, 35	3.91 ± 0.13
DC	9.42 ± 0.22^*^	5.24 ± 0.44	11.73 ± 0.66	12.11 ± 0.41
D + PESB	11.67 ± 0.28	2.37 ± 0.10	47.64 ± 1.48	5.34 ± 0.24
D + GLB	12.13 ± 0.13	1.39 ± 0.02	90.43 ± 1.56	4.77 ± 0.14

*HOMA-β* homeostatic model β, *HOMA-IR* homeostatic model assessment-insulin resistance, *HbA1c* glycosylated hemoglobin, *NC* Normal rats (control), *PESB* Normal + PESB (100 mg/kg), *DC* Control rats with Diabetes, *D + PESB* Diabetic + 100 mg/kg PESB, *D + GLB*: Diabetic + Glibenclamide. Values were expressed as the mean ± SEM; a: comparison between the control group (NC) and all groups, b: comparison between the diabetic group (DC) and all groups, and c: comparison between diabetic group received PESB and diabetic group received GLB (*p < 0.05, **p < 0.01, and ***p < 0.001)

#### Effect of PESB on lipid report

**Fig. 1:**
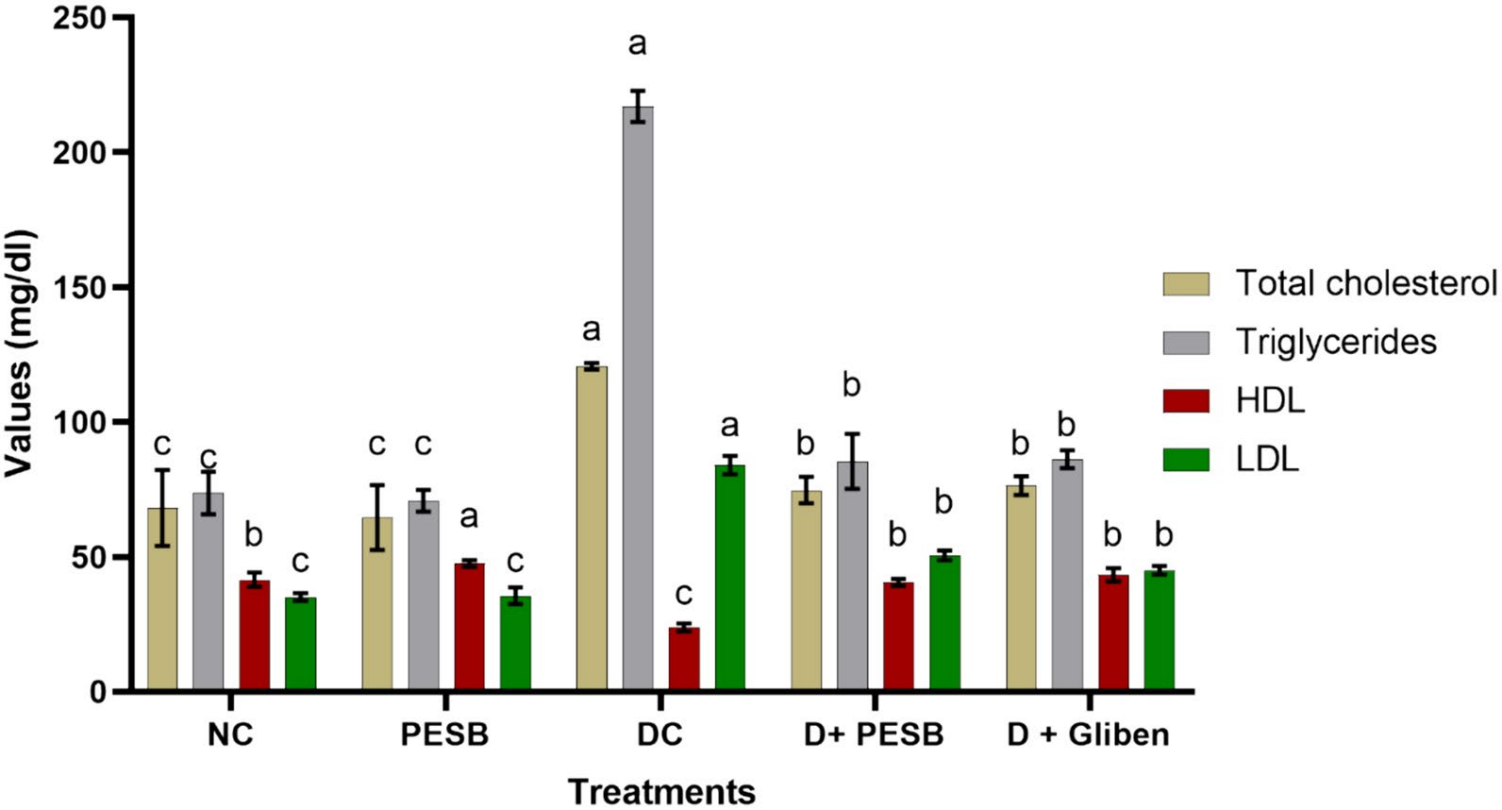
Impact of repetitive daily oral management with the PESB and GLB on blood TC, TG, HDL-C, and LDL-C amounts (mg/dl) in both affected (diabetic) and standard rat (a > b > c > d denote significant difference at p ≤ 0.05 between treatments (groups of rats))

The impacts of GLB and PESB on the levels of blood lipids are illustrated in Fig. 1. Obtained findings showed substantial increases in total cholesterol (TC), triglycerides (TG), and small-density lipoprotein (LDL-C) among diabetic rats. In contrast, the amount of elevated-density lipoproteins (HDL-C) in the blood (plasma) of diabetic rats was substantially decreased. Nevertheless, when these diabetic rats were given daily oral treatment with the SB polyphenolic extract and Glibenclamide, their levels of triglycerides, HDL-C, total cholesterol, and LDL-C remained largely normal. This indicates that these extracts have a beneficial effect by keeping blood lipid levels within healthy limits, even in diabetic rats. These findings suggest a potential treatment to help control cholesterol levels and prevent diabetes-related complications.

Table 5 shows the effects of daily oral PESB and GLB administration on the Coronary Risk Index (CRI), Cardiovascular Risk Index (CVRI), and Atherogenic Index (AI) in both control and diabetic rats. The obtained findings displayed different effects after treatment with both PESB and GLB depending on the studied parameter and group of rats. The untreated diabetic rats with PESB and GLB showed a significant increase in all studied parameters. In contrast, the values of AI, CVRI, and CVRI were statistically similar between non-diabetic and both PESB and GLB-treated diabetic rats.

**Table 5:** Impact of daily oral PESB and GLB treatment on the Atherogenic index (AI), Cardiovascular risk index (CVRI), and Coronary risk index (CRI) in rats with normal and diabetes

Parameters	NC	PESB	DC	D + PESB	D + Gliben
Atherogenic index (AI)	0.84 ± 0.53c	0.75 ± 0.40c	3.53 ± 2.30a	1.25 ± 1.34b	1.04 ± 0.65b
Coronary risk index (CRI)	1.64 ± 0.53b	1.35 ± 0.53b	5.08 ± 0.81a	1.84 ± 0.81b	1.76 ± 1.42b
Cardiovascular risk index (CVRI)	1.77 ± 0.42c	1.48 ± 0.72c	9.14 ± 1.42a	2.11 ± 1.32b	1.99 ± 0.79b

a > b > c > d denote significant difference at p ≤ 0.05 between treatments (groups of rats)

#### Impacts of PESB and GLB on liver enzymes

**Fig. 2:**
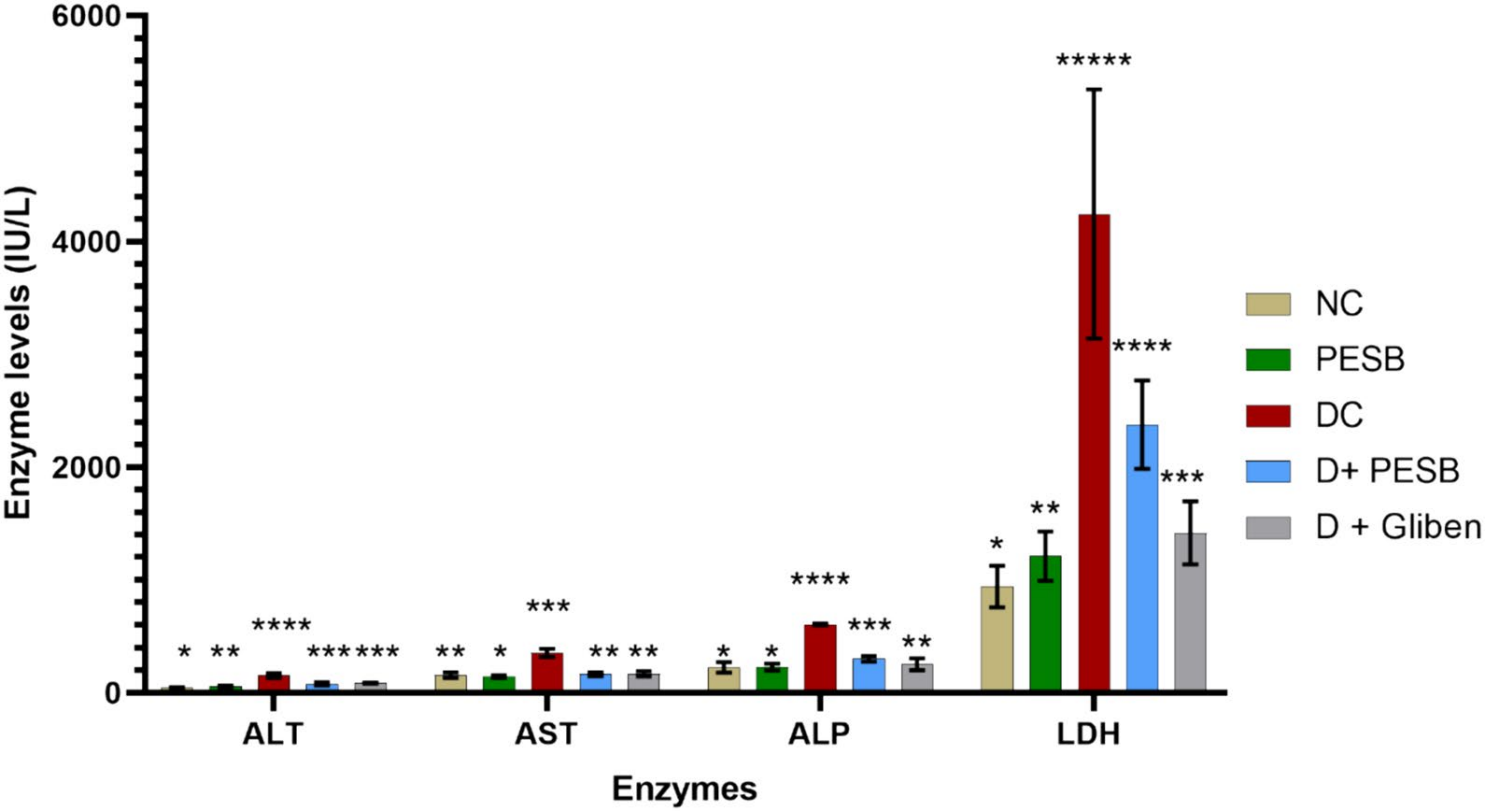
Comparison of the liver enzymes among treated groups of rats (**** > *** > ** > *(significant difference)

During laboratory experiments, we investigated the impacts of the studied extract (PESB) on liver enzyme levels in both diabetic and non-diabetic rats, and the obtained effects are portrayed in Fig. 2. The obtained effects were variable depending on the treatment and group of rats. Based on the analysis of the results, all studied enzymes (AST, ALT, ALP, and LDH) from the liver were substantially augmented in basic groups of diabetic rats (untreated), which indicated liver damage. In contrast, the levels of liver enzymes were decreased in diabetic groups cured with PESB and GLB. The lower level of the enzymes in the liver suggests protective effects from the used extract against liver damage associated with diabetes.

#### Effects of PESB and GLB on renal biomarkers

During laboratory experiments, we investigated the impacts of studied extracts (PESB) and Glibenclamide on the kidneys of diabetic and normal rats to manage kidney dysfunction, which is a widespread complication allied with diabetes (type 2). The obtained results are presented in Tables 6 and 7. The results showed that the untreated group (diabetic rats) had higher levels of creatinine, uric acid, and urea in their blood than normal rats. These substances are indicators of renal dysfunction. On the other hand, blood levels of total protein, potassium, sodium, and chloride did not show a significant change (*p* < 0.001) between diabetic and non-diabetic rat groups, suggesting that these parameters were not directly affected by type 2 diabetes in this study context.

**Table 6:** Impact of repetitive daily oral administration with PESB and GLB on urinary renal parameters in both affected (diabetic) and standard rats

	NC	PESB	DC	D + PESB	D + Gliben
Urea (mg/l)	60.05 ± 0.50b	60.13 ± 0.99c	490.76 ± 9.21a	220.95 ± 2.82b	230.84 ± 3.38b
Uric acid (mg/l)	10.85 ± 2.03c	12.52 ± 3.87c	90.12 ± 8.99aa	25.13 ± 2.41b	28.77 ± 9.41b
Creatinine (mg/l)	3.53 ± 1.87c	4.01 ± 1.08c	134.98 ± 43.14a	48.72 ± 12.78	52.34 ± 7.27b
Sodium (mmol/l)	57.25 ± 8.73b	42.25 ± 15.81c	142.00 ± 5.35a	63.50 ± 4.79b	58.25 ± 7.80b
Potassium (mmol/l)	46.66 ± 11.32d	46.35 ± 13.28d	158.16 ± 19.19a	78.77 ± 8.82b	68.48 ± 12.54c
Chloride (mmol/l)	71.4 ± 7.62c	76.75 ± 5.26c	250.025 ± 11.32a	107.52 ± 1.91b	103.30 ± 24.88b

a > b > c > d denote significant difference at p ≤ 0.05 between treatments (groups of rats)

**Table 7:** Impact of repetitive daily oral administration with PESB and GLB on blood renal parameters in both affected (diabetic) and standard rats

	NC	PESB	DC	D + PESB	D + Gliben
Urea (mg/dl)	42.5 ± 2.64c	41.475 ± 1.18c	67 ± 1.41a	46.5 ± 5.04c	50.35 ± 4.25b
Uric acid (mg/l)	24.25 ± 2.87b	24.25 ± 2.21b	38 ± 3.55a	34.25 ± 4.03a	36.5 ± 4.79a
Creatinine (mg/dl)	0.57 ± 0.03c	0.6 ± 0.08c	0.91 ± 0.05a	0.7625 ± 0.08b	0.82 ± 0.05b
Total protein (g/l)	71.45 ± 1.84b	71.525 ± 3.53b	76.525 ± 2.54b	79.6 ± 4.54a	75.775 ± 2.84b
Sodium (mmol/l)	145.75 ± 2.21a	145.5 ± 2.64a	146.75 ± 3.30a	145 ± 2.58a	145.5 ± 1.91a
Potassium (mmol/l)	7.35 ± 0.46a	7.4125 ± 1.11a	6.875 ± 0.11a	6.5775 ± 0.72a	4.9325 ± 0.27b
Chloride (mmol/l)	102.87 ± 8.33a	96.375 ± 8.32a	98.75 ± 10.55a	93.125 ± 2.86b	97.8 ± 5.27a

a > b > c > d denote significant difference at p ≤ 0.05 between treatments (groups of rats)

Normal rats who received PESB revealed no substantial difference in renal function markers compared to untreated normal rats in total protein, potassium, chloride, and sodium levels. Levels of creatinine, urea, and uric acid in the blood increased in the group of rats with diabetes compared to normal rats. The group of diabetic individuals cured with the extract (100 mg/kg PESB) showed a significant increase in renal markers. Analogous effects were also recorded in the group of rats managed with Glibenclamide.

The induction of type 2 diabetes caused an expansion in levels of creatinine, uric acid, and urea in the urine. In addition, it also resulted in increased urinary excretion of chloride, sodium, and potassium. These changes in the urine components indicate renal dysfunction.

#### Body weight

**Fig. 3:**
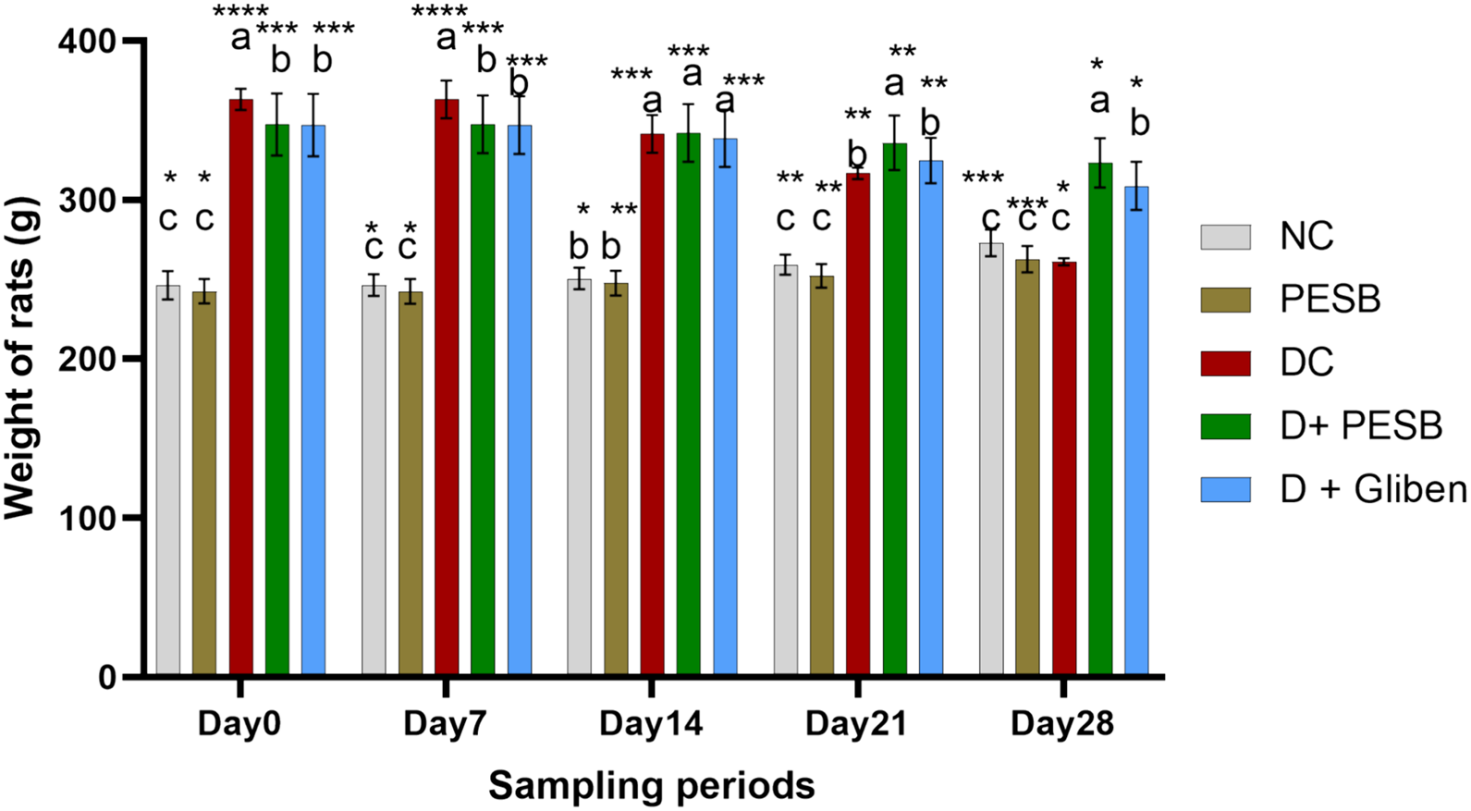
Effects of daily oral PESB and GLB treatment on normal and diabetic rats' body weights (a > b > c > d (significant difference): comparison between experimental periods for the same group of rats)

The effects of daily oral PESB and GLB treatment on the body masses of normal and diabetic rats are illustrated in Fig. 3. Depending on the treatment utilized and the group of rats, the results obtained demonstrated varying effects. Results from the two-factor repeated variance analysis showed that beginning in week 5, all groups fed an HCD diet experienced a substantial rise in body weight in a time-reliant-manner (p < 0.01).

Prior to the administration of polyphenolic extract (PESB) and GLB, or alloxan intervention, the rats in the HCD diet groups (3–5) weighed an average of 150.68 g, significantly (p < 0.01) more than the control groups (1–2) (average 46.5 g). But soon after receiving an alloxan injection and receiving PESB and GLB treatment, the diabetic rat groups significantly reduced their weight, but the normal rat groups' weight increased (p < 0.01). Figure. 5 (weeks 14–17) depicts weight gain following a 4-week intervention. The findings of the variance analysis showed that the diabetic rats (DC, D + PESB, and D + GLB) lost considerably (p < 0.0001) more weight than the non-diabetic groups after the therapy (PESB and GLB) for a period of 4 weeks, weighing −102.27, −24.34, −24.17, and −38.25 g, respectively. Following a 4-week course of management, the weight loss in diabetic groups administered with PESB (100 mg/kg) and GLB was notably less than that of the matching untreated rats (p < 0.01).

#### Impact of PESB and GLB on the tissues

**Fig. 4:**
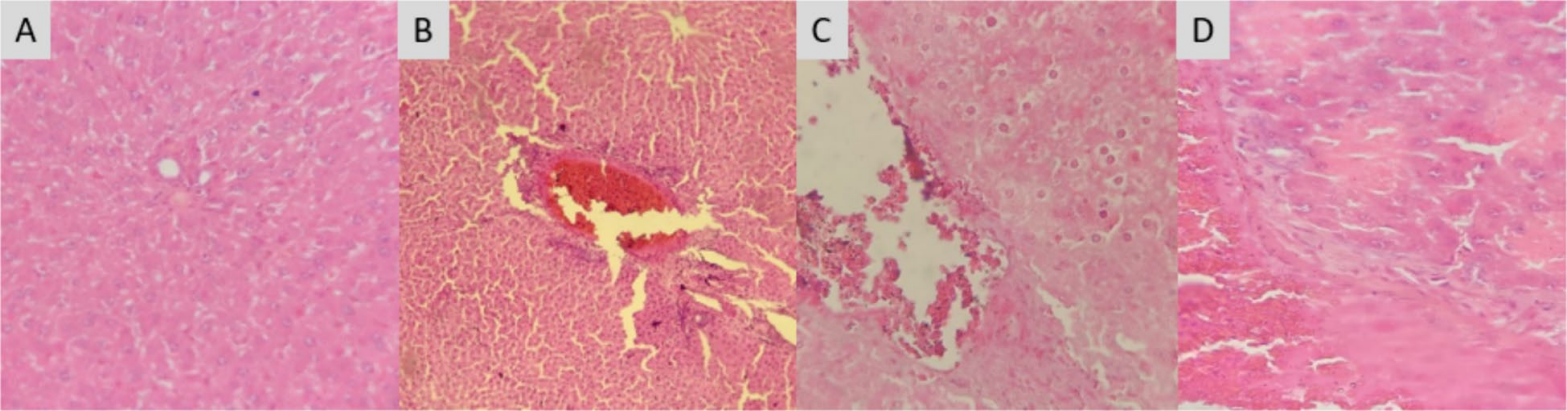
Influence of PESB and Glibenclamide on the tissues of the liver in groups with and without diabetic. **A** Normal control group NC; **B** group of untreated diabetic rats (DC); **C** PESB-treated diabetic group(D + PESB); **D** glibenclamide-treated diabetic rats (D + GLB) (HE 400×)

**Fig. 5:**
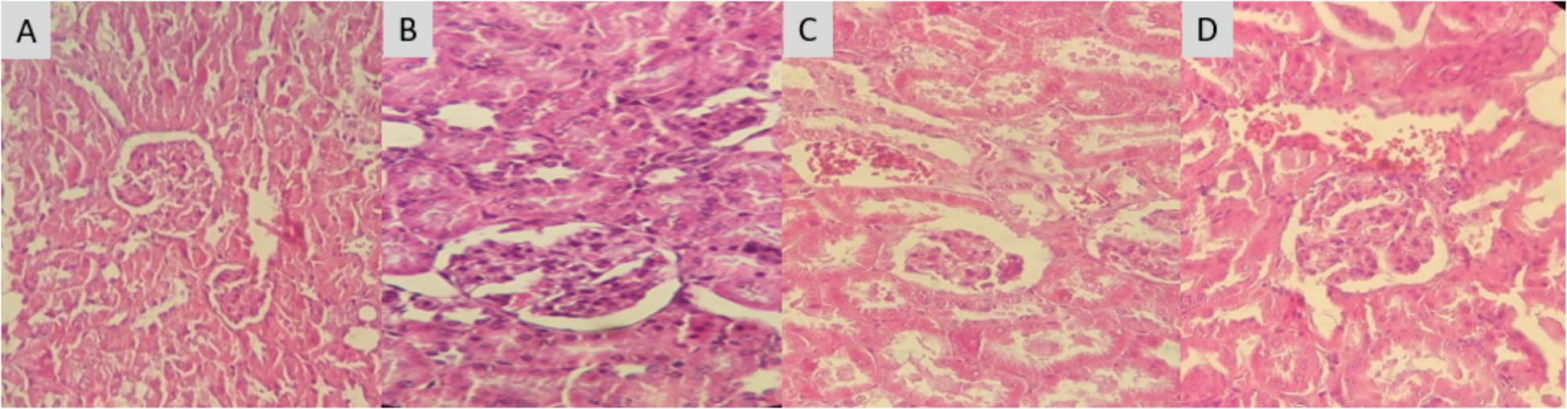
Influence of PESB and Glibenclamide on the tissues of the kidney in rats with and without diabetic. **A** Normal control group NC; **B** group of untreated diabetics (DC); **C** PESB-treated diabetic group (D + PESB); **D** glibenclamide-treated diabetic rats (D + GLB) (HE 400×)

**Fig. 6:**
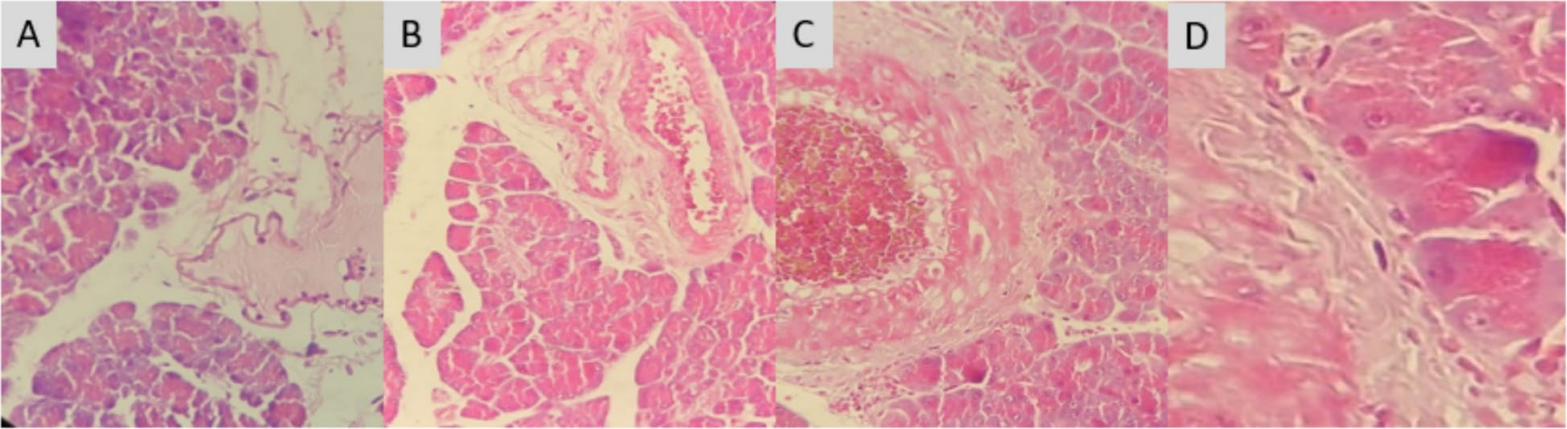
Influence of PESB and Glibenclamide on the tissues of the pancreas in rats with and without diabetic. **A** Normal control group NC; **B** group of untreated diabetics (DC); **C** PESB-treated diabetic group (D + PESB); **D** glibenclamide-treated diabetic rats (D + GLB) (HE 400×)

Hematoxylin–eosin-colored microscopic pictures of rats' liver, kidney, and pancreas from the control and experimental groups are displayed in Figs. 4, 5 and 6. Oral PESB-treated diabetic groups displayed a meaningful improvement in the organization of tissues as compared to untreated diabetic groups. Figure 6 displays the results of the liver's histology examination.

The liver had a normal microscopic architecture in the livers of normal, non-diabetic groups of rats (i.e. Figure. 4A), with hexagon-shaped Pacinian lobules centered around the major vein.

Conversely, in the untreated diabetic rat group (Fig. 4B), there was liver structural disorder and congestion in the central vein, which could result in serious injury and necrosis. Conversely, in the untreated diabetic rat group (Fig. 4B), there was liver structural disorder and congestion in the central vein, which could result in serious injury and necrosis. When polyphenolic extract is administered, the liver's morphology approaches that of the normal group (Fig. 4C). Likewise, therapy with glibenclamide led to mild dilation of the gate vein, reduced congestion in the hepatic arteries, and mild liver disease (Fig. 4D).

The kidneys of untreated diabetic rats (Fig. 5B) underwent significant alterations when viewed under a microscope in comparison to the control group (Fig. 5A). A decrease in the volume of the Bowman capsule, coupled with expansion of the renal veins and structural kidney disease, characterize these changes. Conversely, the renal tissues of the subjects PESB-treated (Fig. 5C) and Glibenclamide-treated (Fig. 5D) exhibit notable resistance to lesions caused by alloxan.

The histological analysis of pancreatic tissue in diabetic rats, as demonstrated in Fig. 6, reveals that the rats have an injured pancreas and that the islets have withered with substantial obliteration of beta cells when compared to rats in control groups (Fig. 6B). On the other hand, pancreatic organization with moderate inflammation is markedly improved by oral treatment of PESB (Fig. 6C). The tissues of the pancreas in diabetic groups treated with Glibenclamide (Fig. 6A) display an organization similar to that of normal rats' pancreases (Fig. 6D).

## Discussion

### Hypoglycemic parameters

Currently, many reports have addressed the capacity of therapeutic vegetations to reduce the impacts of diabetes in clinical assays (Franco et al. 2018; Shahin D. H. et al. 2022; Arifah et al. 2022). These investigations demonstrated the positive effects of chemical compounds in medicinal plant derivatives to manage the impacts of type 2 diabetes (Unuofin and Lebelo 2020; Blahova et al. 2021; Balogun et al. 2022; Paşayeva et al. 2022). In the current study, we evaluated the beneficial influences of PESB from *S. blancoana* subsp*. mesatlantica* in the management of hypercaloric-fed and small-dose alloxan-tempted diabetes of type 2 in rats. Equally, we explored the chemical compounds and antioxidant activity of the extract to explain its biological activity. The obtained results showed new data on the positive effects of the extracts on reducing the impacts of diabetes in treated rats. The variety of chemical combinations and antioxidant properties of the extracts control these effects. As far as we are aware, these findings are the first to estimate the anti-diabetic properties of this substance derived from *S. blancoana* subsp. *mesatlantica*, providing new opportunities for the investigation of diabetes treatment.

Our findings demonstrate that 12 weeks of continuous high-calorie diet (HCD) exposure led to statistically significant increases in insulin in serum, and decreases in HbA1c, and glucose levels in the blood. Apart from a markedly lowered HOMA-IR score and increased HOMA-β score, which validate the improvement of diabetes (type 2) and resistance to insulin. This finding is consistent with the results in the bibliography (Wang et al. 2020; Jafari et al. 2021; Assadi et al. 2021). The coexistence of hyperglycemia and hyperinsulinemia is a key diagnostic sign of DT2 (Soliman 2016; Moke et al. 2023). Prolonged hyperinsulinemia induces negative phosphorylation control of insulin receptors and a changeable reduction in the activity of tyrosine receptor kinase, thereby guiding to insulin resistance supplementary to type 2 diabetes (Kanety et al. 1994; Catalano et al. 2014).

Blood glucose levels were significantly reduced over the course of 28 days by daily oral medication with 100 mg/kg of *S. blancoana* polyphenolic extract (PESB); they were 401.13 mg/dl on day 0 and 176.50 mg/dl on day 30 after intervention. Furthermore, in diabetic rats (with type 2), this intervention improved the HOMA-IR and HOMA-score and raised insulin levels. The anti-diabetic effects of PESB are supported by the observed reductions in hemoglobin HbA1c and levels of fasting sugar in the blood, as well as the upgrade in plasma insulin levels. The efficacy of PESB in reducing blood sugar levels is associated with findings from earlier research on diabetic Wistar rats produced by streptozotocin (STZ) (Eidi and Eidi 2009), Sprague–Dawley Type 2 diabetes (T2D), diabetic rats created by STZ plus a high-calorie diet (Huang et al. 2015), and dietetic rabbits induced by STZ (Mokogwu et al. 2022).

Studies on *S. officinalis* leaves shown a hypoglycemic effect in rats whose streptozotocin-induced hyperglycemia (Eidi et al. 2005; Eidi and Eidi 2009). A study found that an infusion of *S. officinalis* tea was equally as metformin, a medication that is frequently used to remedy type 2 diabetes (Christensen et al. 2010). Additionally, a multitude of investigations have showcased the exceptional anti-diabetic capabilities of sage extracts, emphasizing their potent inhibition of α-glucosidase and α-amylase (Bahadori et al. 2017a, b).

The hypoglycemic effects of sage extracts containing components like carnosic substance, phenolic acids, rosmarinic acid, and flavonoids or their derivatives have been shown in previous research (Wang et al. 1998; Rustaiyan et al. 1999; Durling et al. 2007). Additionally, it has been proposed that sage's hypoglycemic impact may be attributed to the existence of phenolic chemicals (Eidi et al. 2005). Regarding this, a study by Kalaycıoğlu et al. (2018) assessed the α-glucosidase activity-inhibiting potential of extracts from 14 Turkish sage species. With the lowest IC_50_ values of 17.6 followed by 25.9 μg/mL, respectively, *Salvia aucheri* var. aucheri and *Salvia adenocaulon* samples demonstrated the most marked repressive effects on the activity of α-glucosidase among the considered samples.

The fraction of polyphenolic acid from *S. miltiorrhiza* (i.e. contains SalB at 53.6%), was shown to dramatically lower glucose in blood, triglyceride, and total cholesterol amounts while improving sensitivity to insulin in rats with type 2 diabetic in a prior study by Huang et al. (2016).

### Effect on liver enzymes

Being the foremost metabolic structure of the body, the liver is imperative to maintaining the proper ratio of fats and carbohydrates (Saravanan and Pari 2003). Enzymes called AST and ALT play a direct role in the transformation of amino acids into ketone acids (Whitehead et al. 1999). Elevated AST and ALT activity in plasma is an indicator of liver impairment caused by diabetes. Hyperglycemia raises malondialdehyde levels, decreases antioxidant levels, and causes oxidative damage to the liver (Behrouj et al. 2019). Our results highlight that alloxan has a hepatotoxic effect, which could be caused by the reactive oxygen species (ROS) that diabetes produces. They also suggest that the increased serum levels of the hepatic enzymes ALT, AST, ALP, and LDH are likely initiated by the bloodstream’s introduction of these enzymes from the liver's cytosol (Laaroussi et al. 2020). Another explanation for tissue transaminase rise could be a rise in protein catabolism associated with urea and gluconeogenesis. Aspartate aminotransferase activity typically rises in tandem with elevated alanine aminotransferase activity, which indicates hepatic cell injury.

Moreover, because of their chemical makeup, flavanones, a particular class of flavonoid, have the potential to be employed as antidiabetic drugs (Ortiz-Andrade et al. 2008; Youssef et al. 2014). The major flavanone known as naringin, which we found in our samples of *S. blancoana,* and which is primarily found in citrus fruits, has been shown to effectively expand the glycemic status in diabetic mice by modulating the expression (gene) of the enzymes tangled in glucose homeostasis and partially regulating cholesterol metabolism. It also reduces oxidative concern and the creation of inflammatory cytokines (i.e., pro-inflammatory) in rats affected by diabetes stimulated by high-fat diet and streptozotocin (Ahmed et al. 2010).

Previous studies conducted in 2017 on rats affected by type 2 diabetes brought on by streptozotocin (STZ) and nicotinamide (NA) assessed the anti-hyperglycemic, antihyperlipidic, and antioxidant effects of navel (a variety of orange) bark hydroethanol extracts and their constituent flavonoids, including naringenin and naringin. In type 2 diabetic rats given NA/STZ, these substances found in navel orange bark extracts (i.e., naringenin and naringin) have demonstrated convincing anti-diabetic actions. Further, their competence to degrade insulin resistance and their insulinotropic qualities are credited with these effects, which are most likely caused by increased expression of GLUT4, adiponectin, and insulin receptors in adipose structures (Ahmed et al. 2017). In a 2016 study, Ren et al. found that phenolic compounds, specifically naringin and apigenin, had similar positive effects on rats affected by type 2 diabetes emitted by streptozotocin and a high-fat diet. In rats with DT2, these substances enhanced endothelial dysfunction, glucose, and lipid metabolism, possibly by lowering oxidative stress and inflammation (Ren et al. 2016). Numerous investigations have demonstrated a connection between the phenolic chemicals in sage and its hypoglycemic impact. Specifically, our sample included substances including apigenin, cinnamic acid, and syringic acid (Table 2), all of which significantly reduced blood sugar levels (Cazarolli et al. 2009; Kasetti et al. 2012; Muthukumaran et al. 2013).

Glucose binds irreversibly to the N-terminal valine of the beta binding of hemoglobin when sustained hyperglycemia is present. Further, glycation in additional places, such as the beta-chain of the lysine on alpha-chain places, may be important at higher glycation levels. The rats treated with PESB and GLB showed lower levels of hemoglobin, suggesting that the bioactive compounds in these products may have prevented the glycation process (Vellai et al. 2018). It is known that prolonged exposure to excessive glucose levels inhibits the production of insulin and is correlated with the expansion of peripheral insulin resistance. The particular action of alloxan on pancreatic beta cells results in an evidenced decline in blood insulin amounts in diabetic groups of rats. However, a significant increase in insulin levels was observed in diabetic rat groups that received EPSB, demonstrating the ability of the polyphenolic extract of *S. blancoana* to stimulate insulin production and preserve tissues.

The chemicals included in the plant extract may be the cause in the boost of insulin in plasma investigated in diabetic groups administered sage extract. These substances may either increase the release of insulin or shield functional, undamaged β cells from more damage, therefore preserving their activity and ability to produce insulin. The restoration of normal blood sugar levels may mitigate the glucotoxicity that damages β-cells and protect β cells, at least partially.

For the cure of diabetes, extensive examination is being conducted to find plant- or synthetic-derived alternatives to insulin, secretagogues, or sensitizers, even if insulin has become one of the most significant therapeutic drugs in medicine (Eidi and Eidi 2009).

It is important to treat diabetes type 2, which is characterized by progressive loss of beta cells and decreased output of insulin as a result of resistance to insulin. The resistance and function of beta cells are computed by the homeostasis evaluation (HOMA-IR). Through the Randle cycle, a high-fat regime causes insulin resistance, which hinders the transfer of insulin-stimulated glucose and lowers beta-cell activity (Randle et al. 1963). PESB treatment dramatically reduces glucolipotoxicity by improving beta cell activity and lowering HOMA-IR.

Overall, the study indicates that the antihyperglycemic effect of sage is attributed to the interaction between the phenolic compounds and the body’s glucose-regulating mechanisms.

However, there was a noticeable drop in these enzymes in both the PESB group and the glibenclamide (GLB) group. These results are in line with what other research has found (Eidi and Eidi 2009). It is conceivable that the extract's phenolic components are what provide the observed protection against liver damage. These substances have a reputation for being anti-inflammatory and antioxidants, which may help shield the liver from the damaging effects of diabetes. Due in part to their effects on the AMPK pathway, other earlier research has shown the potential anti-diabetic qualities of natural substances such as resveratrol, curcumin, and berberine (Lakshmanan et al. 2011; Do et al. 2012; Xu et al. 2014).

These findings demonstrate how crucial it is to treat type 2 diabetes by focusing on the AMPK pathway. Huang and colleagues’ recent work (Huang et al. 2016) assessed the impact of SalB, a chemical present in *S. miltiorrhiza* Bunge, on diabetes type 2 in male mice (C57BL/KsJ-db/db). B-Sal increased insulin tolerance, restored liver and muscle function, and decreased blood sugar, insulin, triglycerides, and fatty acids in these diabetic rats. These results are like what metformin has been shown to produce. Moreover, administration of SalB led to elevated expression of glycogen synthase and glucose 4 carrier proteins (i.e., GLUT4) in the muscles of the skeleton, as well as phosphorylated expression of p-AMPK (AMP-activated protein kinase) in the skeletal muscle and liver. Additionally, it led to an upregulation of peroxisome proliferator-triggered alpha receptor (PPARα) and acetyl CoA carboxylase (p-ACC) protein expression in the liver.

Shahabaddin et al*.* conducted a study in 2021 to investigate the potential preventive effects of hydroethanol fruit extract (HECS) derived from *Capparis spinosa* on type 2 diabetes and oxidative stress in diabetic rats administered a high-fat diet (HFD) along with a low dosage of streptozotocin (STZ). The results showed that by increasing the levels of antioxidant-using enzymes and lowering lipid peroxidation in liver tissues, HECS significantly reduced oxidative stress and glucose intolerance in diabetic rat. Furthermore, in the expression of mRNA in diabetic rats, HECS considerably reduced the activity of hepatic phosphoenolpyruvate carboxykinase (PEPCK), raised that of acetyl coenzyme A carboxylase, and somewhat lowered the PEPCK transactivator and nuclear factor-4α of hepatocytes (HNF-4α) (Assadi et al. 2021).

### Effect on renal biomarkers

In comparison to the control group (rats), untreated diabetic rats demonstrated a statistically significant rise in levels of creatinine, urea, and uric acid in both serum and urine. Additionally, despite stable blood levels of sodium, chloride, and potassium, there was an increase in these elements in the urine of diabetic rats, indicating renal damage associated with hyperglycemia (Kim et al. 2017).

According to these rulings, oxidative damage is the main cause of renal toxicity caused by diabetes. Supplementation with phenolic acid appears to be a useful tactic to mitigate this toxicity and shield kidney function from the damaging effects of diabetic hyperglycemia (Forbes et al. 2008).

Following treatment with sage polyphenolic extract, these indices returned to normal, demonstrating improved kidney function and decreased metabolic disruptions in multiple pathways, including protein and nucleic acid metabolism. The extract’s potential to improve blood sugar regulation may be the cause of this improvement.

Metabolic problems associated with diabetes are typified by raised triglyceride and cholesterol levels, elevated lipid peroxidation, and increased xanthine oxidase action (Madianov et al. 2000). Diabetes-related protein glycation can also result in muscle atrophy, an increase in purine releases the primary source of uric acid, and an increase in xanthine oxidase activity (Anwar and Meki 2003). These results are consistent with earlier research (Eidi and Eidi 2009), highlighting the useful role that sage extract plays in helping diabetic rats' metabolisms return to balance.

### The effect on lipid balance

One important risk factor for cardiovascular disease is diabetic dyslipidemia (Solano and Goldberg 2006). Our discoveries show that the lipid profile recorded in diabetic rats is significantly disrupted, with significant prominent levels of cholesterol, LDL-C, and triglycerides in plasma compared to those in control rats, and HDL-C significantly lower. This condition raises the risk of oxidative damage at rates affected by diabetes type 2, which can result in prolonged cardiovascular illness (CVD) and atherosclerosis (Molitch 2006). LDL oxidation is frequently connected to atherosclerosis (Matsuura et al. 2008).

Hypertriglyceridemia in diabetes mellitus can be caused by elevated VLDL (very low-density lipoprotein) synthesis and modified triglyceride-rich particle breakdown. Insulin resistance and insufficiency both have an impact on lipoprotein lipase, the primary enzyme that removes lipids from the bloodstream. Moreover, the abnormally high concentration of plasma lipids in individuals with diabetes primarily results from the increased release of free fatty acids, a process in which insulin is necessary to inhibit hormone-sensitive lipase (Garg 1994). A reduction in the HDL-C level may also result from a decline in the activity of lecithin cholesterol acyl transferase (Chandramohan et al. 2010).

Sage polyphenolic extract treatment in diabetic rats helped return normal lipid levels, supporting earlier findings (Kianbakht et al. 2016). Herbal extracts have been shown to have hypolipidemic properties in earlier studies (Ji and Gong 2008; Assadi et al. 2021). This ability to lower cholesterol can be explained by a variety of factors, including a decrease in intestinal cholesterol absorption, a decrease in cholesterol production, an increase in LDL receptors and their absorption, and an increase in LCAT activity (Eddouks et al. 2005). Moreover, diminished greasy acid synthesis, enhanced catabolism of LDL, elevated lipase in tissues and LCAT activity, and reticence of acetyl-CoA carboxylase could all contribute to hypotriglyceridizing effects (Eddouks et al. 2005).

PESB's ability to regulate hyperglycemia may be related to its effect on hypercholesterolemia and hypertriglyceridemia in diabetics. This is in line with the finding that total lipoprotein and very low-density lipoprotein triglyceride concentrations are significantly influenced by the degree of glycemic control (Laakso and Kuusisto 2003).

The occurrence of flavonoids, saponins, and other phytochemical elements in common sage has been noted in previous investigations (AYAT et al. 2009). This richness raises the possibility that the combined effects of these chemicals may account for Salvia's hypoglycemic effect (Tiwari and Rao 2002). Similar metabolic profiles are shared by Chinese sage (*S. miltiorrhiza*), whose extracts have been found to lower triglyceride and cholesterol levels (Christensen et al. 2010). Studies have shown that the gamma receptor that is activated by peroxisome proliferators is a regulator of genes implicated in the metabolism of glucose, fats, and energy is activated by the *S. officinalis extract*. This lowers blood triglycerides, improves the ratio of high-density to low-density lipoprotein, and lessens insulin resistance (Christensen et al. 2010). The extracts' ability to stop LDL cholesterol from oxidizing has also been demonstrated in other earlier research, which helps to lower the risk of cardiovascular disease (Sá et al. 2009).

### Effect on body weight

Our findings show that, in accordance with earlier studies (Zhang et al. 2015; Kuate et al. 2015; Assadi et al. 2021), the rats in the control groups gained weight continuously over the course of 17 weeks, but the rats in groups 3 to 5, who subsequently developed diabetes, only gained weight when they were on a hypercaloric diet. But following an injection of alloxan, their body weight dropped. Because type 2 diabetes increases fat metabolism and decreases glucose metabolism, weight loss is a common symptom of the disease (Zhang et al. 2015). Weight loss was considerably reduced in rats given PESB treatment, indicating that sage extract protects against hyperglycemic tissue damage. Previous research has demonstrated that *S. officinalis* leaf extracts cause diabetic rats to gain weight (Eidi et al. 2005; Mokogwu et al. 2022).

### Histopathological examination

Considering that complications arising from diabetes primarily affect the liver, kidneys, and pancreas, the histopathological changes observed in these organs among subjects with diabetes are consistent with findings reported in other studies (Hamden et al. 2008; Mohammadi and Naik 2012). Sage extract shows promise as a defense against these DT2-induced changes. The protection observed can undoubtedly be attributed to the bioactive substances and antioxidants found in the polyphenolic extract PESB, such as syringic acid and vanillic acid (Drissi et al. 2004; Shukri et al. 2010).

According to the research conducted by Sun et al. (2012) and Huang et al. (2015) Sal B (salvianolic acid B) has demonstrated its protective power against high glucose-induced toxicity in other studies by demonstrating the antioxidant and protective effects of salvianolic acids in *Salvia miltiorrhiza* L. These researchers found that Sal B inhibits activated caspase-3, limits the liberation of cytochrome C (from the mitochondria) in the cytosol, balances expression in members of the Bcl-2 class, and reduces the expression of the type 1 cancer mortification factor-alpha receptor (Yan et al. 2010). Additionally, other research, including that done by Gao et al. (2012), suggests that using Sal B as a treatment could lessen inflammation brought on by specific harmful substances. This may be explained by its anti-inflammatory properties, which, according to Chen et al. (2011), decrease the manifestation of the pro-inflammatory cytokines IL-1b and TNF-a while enhancing the manifestation of the TGF-b1 and IL-10 anti-inflammatory cytokines.

Kim et al. (2017) found that eucalyptol, a main ingredient in *S. blancoana* subsp. *mesatlantica* essential oil, significantly decreased hyperglycemia, and proteinuria in mice (db/db) when given at an amount of 10 mg/kg (for a period of eight weeks). It also prevented the alterations to the cell adhesion proteins N, E, and P as well as the formation of collagen fibers in the kidneys of diabetics. Eucalyptol repressed the stimulation of proteins implicated in the epithelial-mesenchymal transition, including β-catenin, Snail1, and integrin-1-related kinase (ILK1), in tubular cells displayed to glucose and in kidneys impacted by diabetes, while reversing the expression of glycogen synthase kinase (GSK)-3β. Furthermore, in cylindrical cells exposed directly to glucose, eucalyptol attenuated the enhanced production of TGF-β1, which in turn weakened the stimulation of β-catenin, Snail1, and ILK1. Further, in cylindrical cells exposed directly to glucose and eucalyptol, the genetic suppression of Snail1 also prevented the induction of β-catenin, which increased the expression of GSK-3β (Kim et al. 2017). pointing out that a significant amount of eucalyptol can be found in *S. blancoana* subsp. *Mesatlantica*’s essential oil (Maache et al. 2023).

## Conclusion

This investigation assessed the antioxidant activity and the beneficial effects of *S. blancoana* subsp. *mesatlantica* in the control of type 2 diabetes. Equally, it investigated the chemical constituents of the plant to explain their biological properties. In order to achieve these goals, we utilized the polyphenolic extract (PESB) from the leaves of *S. blancoana subsp. mesatlantica* in rats with hypercaloric-fed and small-dosage alloxan-induced type 2 diabetes. Polyphenolic extract of *S. blancoana* subsp. *mesatlantica* significantly reduced the effects of type 2 diabetes, including the decrease of HbA1c and HOMA-IR and the increase of HOMA-β and plasma insulin. Similarly, the PESB extract reduced the levels of lipids, including LDL-C, total cholesterol, triglycerides, and enzymes of the liver (ALT, AST, ALP, and LDH), in diabetic rats. Further, the PESB treatment significantly reduced the weight of diabetic rats. The histologic observations confirmed the protective roles of polyphenolic extract (PESB) in the tissues of the pancreas, kidney, and liver of diabetic rats. The present study shows that the extract from *S. blancoana* subsp. *mesatlantica* is effective in managing diabetes mellitus because it is rich in a variety of bioactive compounds. Therefore, more inquiry is required to evaluate the safety of the plant. In summary, it can be demonstrated that the traditional usage of *S. blancoana* subsp. *mesatlantica* as an agent for antidiabetic roles is justified, and the polyphenolic extract of this plant has activity analogous to that of the customary antidiabetic medication, glibenclamide.

## Data Availability

All data generated or analyzed during this study are included in this published article and its Additional files, further inquiries can be directed to the corresponding authors.

## Abbreviations

TD2: Type 2 diabetes
PESB: Polyphenolic extract from *Salvia blancoana *subsp*. mesatlantica*
HPLC–DAD: High-performance liquid chromatography
TAA: Total antioxidant activity
DPPH: 2.2-Diphenyl-1-picrylhydrazyle
RP: Reducing Power
TFC: Flavonoid content
GLB: Glibenclamide
IDF: International Federation of Diabetes
HCD: High-calorie diet
NC: Normal control
DC: Diabetic control
HbA1c: Glycated hemoglobin
AST: Aspartate aminotransferase
ALP: Alkaline phosphatase
ALT: Alanine aminotransferase
LDH: Lactate dehydrogenase
TG: Triglycerides
TC: Total cholesterol
HDL-C: High-density lipoproteins
LDL-C: Lipoprotein with low-density
Na^+^: Sodium
K^+^: Potassium
Cl^−^: Chloride
RIA: Insulin radio immunoanalysis
HOMA-IR: Homeostatic model assessment-insulin resistance
HOMA-β: Homeostasis model assessment of β-cell function
CVRI: Cardiovascular risk Index
AI: Atherogenic index
CRI: Coronary artery risk index
HE: Hematoxylin–eosin
STZ: Streptozotocin
SalB: Salvianolic acid B
ROS: Reactive oxygen species
NA: Nicotinamide
AMPK: Activated protein kinase
PPARα: Peroxisome proliferator-triggered alpha receptor
p-ACC: Acetyl CoA carboxylase protein
HECS: Hydroethanol extract from *Capparis spinosa*
PEPCK: Phosphoenolpyruvate carboxykinase
HNF-4α: Nuclear factor-4α of hepatocyte
CVD: Cardiovascular disease
VLDL: Very low-density lipoprotein
ILK1: Integrin-1-related kinase
GSK: Glycogen synthase kinase
