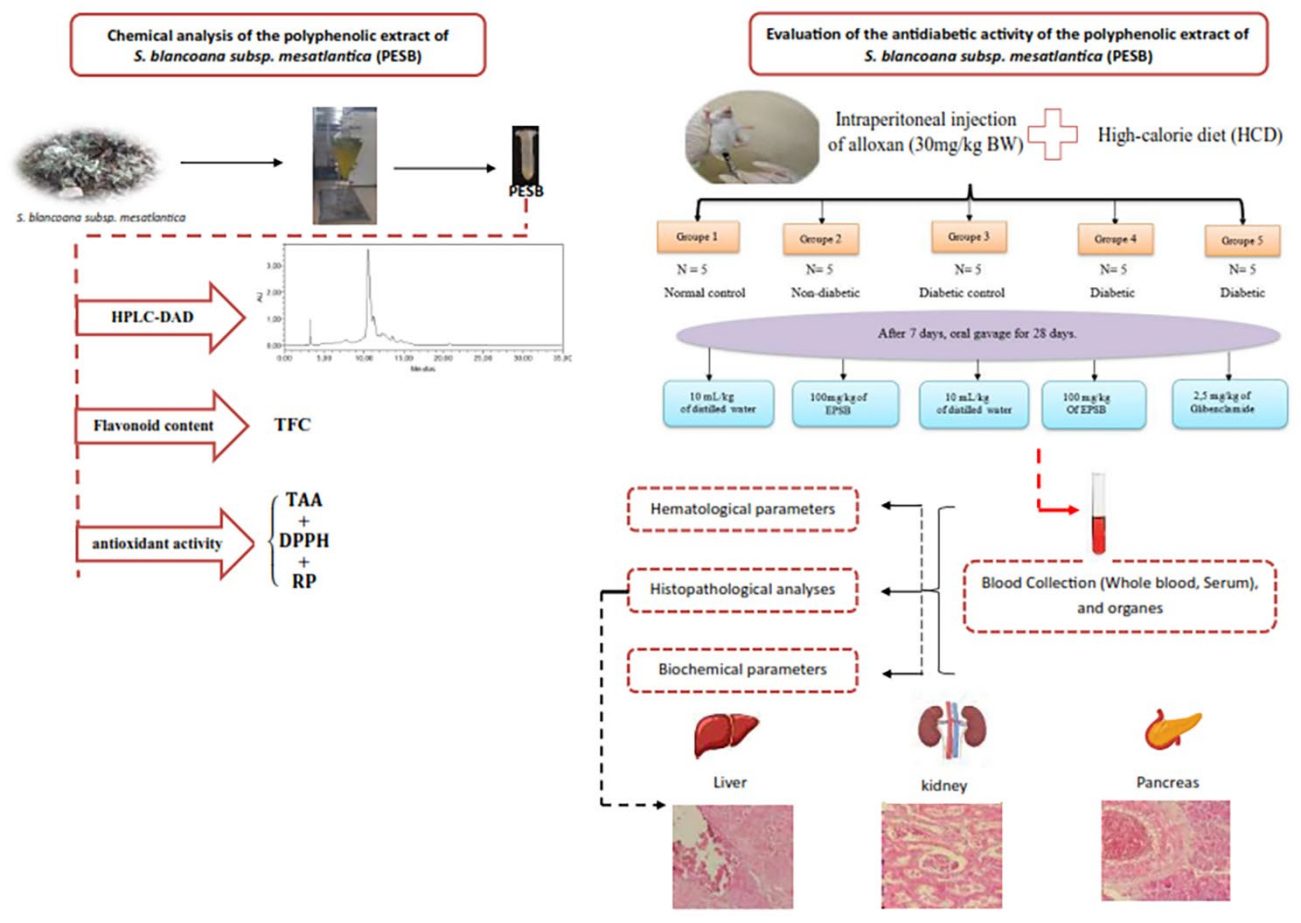# Correction: The antioxidant, antidiabetic, and antihyperlipidemic efects of the polyphenolic extract from Salvia Blancoana subsp. Mesatlantica on induced diabetes in rats

**DOI:** 10.1186/s40643-024-00780-6

**Published:** 2024-07-18

**Authors:** Souad Maache, Hassan Laaroussi, Najoua Soulo, Ghizlane Nouioura, Nabil Boucetta, Mohammed Bouslamti, Hamza Saghrouchni, Yousef A. Bin Jardan, Samir Ibenmoussa, Mohammed Bourhia, Badiaa Lyoussi, Ilham Elarabi

**Affiliations:** 1 Laboratory of Natural Substances, Pharmacology, Environment, Modeling, Health, and Quality of Life (SNAMOPEQ), Faculty of Sciences Dhar El Mahraz, Sidi Mohamed Ben Abdellah University, Fez, Morocco; 2 Medical Analysis Laboratory Sais, Fez, Morocco; 3 Department of Biotechnology, Institute of Natural and Applied Sciences, ?ukurova University, Balcal?, Adana, 01250 Türkiye; 4 Department of Pharmaceutics, College of Pharmacy, King Saud University, P.O. Box 11451, Riyadh, Saudi Arabia; 5 Laboratory of Therapeutic and Organic Chemistry, Faculty of Pharmacy, University of Montpellier, Montpellier, 34000 France; 6 Laboratory of Biotechnology and Natural Resources Valorization, Faculty of Sciences, Ibn Zohr University, Agadir, 80060 Morocco; 7 Laboratory of Chemistry-Biochemistry, Environment, Nutrition, and Health, Faculty of Medicine and Pharmacy, University Hassan II, B. P. 5696, Casablanca, Morocco

**Correction: Bioresources and Bioprocessing (2024) 11:62.** 10.1186/s40643-024-00769-1

In this article, the incorrect graphical abstract has been published and it has been corrected in this correction and the original article has been corrected.

The correct version of graphical abstract is given below,

**Graphical abstract**

**Figure 1:**